# Supplementary material for: The interactome of intact mitochondria by cross-linking mass spectrometry provides evidence for coexisting respiratory supercomplexes
Source: Mol Cell Proteomics. 2017 Dec 8;17(2):216–32. doi: 10.1074/mcp.RA117.000470 (PMC5795388; doi:10.1074/mcp.RA117.000470)
Supplement: Supplemental Data [file supp_RA117.000470_133922_0_supp_23978_fzffwf.zip › spectra_annotation/mito_DR_spectra_annotation/496-1-2-1-18-1.pdf]

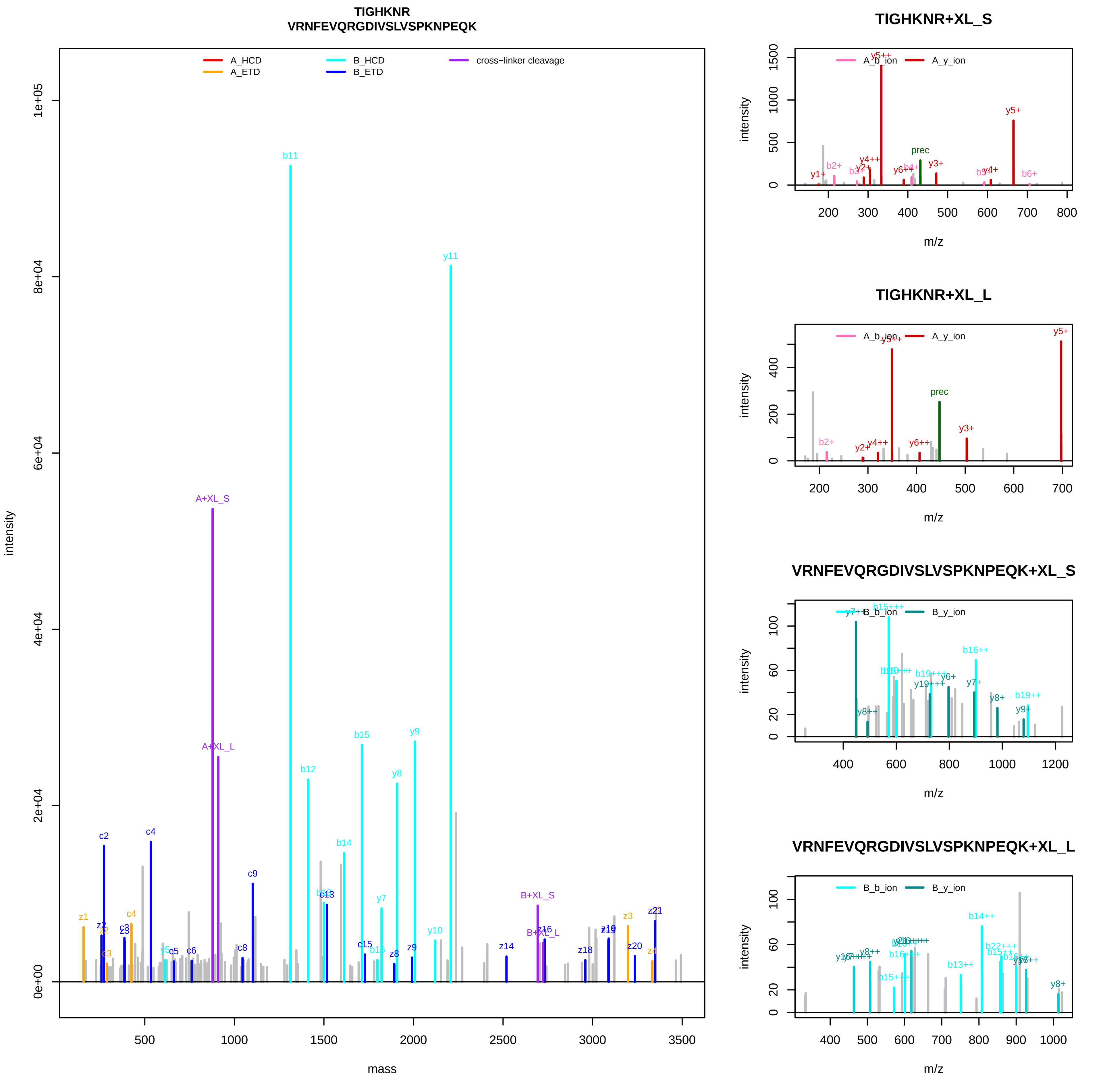

TIGHKNR
VRNFEVQRGDIVSLVSPKNPEQK
A_HCD
A_ETD
B_HCD
B_ETD
cross−linker cleavage
intensity
mass
0e+00
2e+04
4e+04
6e+04
8e+04
1e+05
500
1000
1500
2000
2500
3000
3500
b11
y11
A+XL_S
A+XL_L
B+XL_S
B+XL_L
b12
b13
b14
b15
b16
y5
y7
y8
y9
y10
c2
c3
c4
c5
c6
c8
c9
c13
c15
z1
z2
z3
z4
z8
z9
z14
z16
z18
z19
z20
z21
TIGHKNR+XL_S
TIGHKNR+XL_L
VRNFEVQRGDIVSLVSPKNPEQK+XL_S
VRNFEVQRGDIVSLVSPKNPEQK+XL_L
A_b_ion
A_y_ion
B_b_ion
B_y_ion
prec
m/z
